# Ocular Manifestations in a Chinese Pedigree of Familial Amyloidotic Polyneuropathy Carrying the Transthyretin Mutation c.401A>G (p.Tyr134Cys)

**DOI:** 10.3390/genes13050886

**Published:** 2022-05-16

**Authors:** Xiaonan Zhuang, Zhongcui Sun, Fengjuan Gao, Min Wang, Wenyi Tang, Wei Liu, Keyan Wang, Jihong Wu, Rui Jiang, Gezhi Xu

**Affiliations:** 1 Department of Ophthalmology, Eye & ENT Hospital, Fudan University, Shanghai 200031, China; zxn4258@163.com (X.Z.); zhongcui.sun@aliyun.com (Z.S.); gaofengjuan0815@sina.com (F.G.); wangmin83@yahoo.com (M.W.); drtangwy@163.com (W.T.); bfgf2020@163.com (W.L.); drwangky@163.com (K.W.); jihongwu@fudan.edu.cn (J.W.); 2jiang@163.com (R.J.); 2 Shanghai Key Laboratory of Visual Impairment and Restoration, Fudan University, Shanghai 200031, China; 3 NHC Key Laboratory of Myopia, Fudan University, Shanghai 200031, China

**Keywords:** transthyretin, amyloid, arteriovenous passage time, multifocal electroretinography, internal limiting membrane, transforming growth factor-β1

## Abstract

Familial amyloid polyneuropathy (FAP) caused by a genetic mutation in transthyretin (TTR) is an autosomal dominant hereditary disease. The retrospective, observational case series study presents the ocular clinicopathological findings of five cases carrying the TTR mutation c.401A>G (p.Tyr134Cys). Multimodal retinal imaging and electrophysiological examination, Congo red staining and immunohistochemical analysis of specimens, and genetic analyses were performed. Cases 1 and 2 were symptomatic with vitreous and retinal amyloid deposition and poor visual recovery. Case 3 had a symptomatic vitreous haze in the left eye with good postoperative visual recovery. The right eye of case 3 and the eyes of cases 4 and 5 were asymptomatic. Thicker retinal nerve fiber layer, retinal venous tortuosity with prolonged arteriovenous passage time on fluorescein angiography and retinal dysfunction detected by multifocal electroretinogram occurred even in asymptomatic eyes. Moreover, the internal limiting membrane from patients with FAP was stained positive for Congo red and transforming growth factor-β1. The results highlight the amyloid deposition of mutant TTR in the optic disc and retina, even in the asymptomatic stage. The deposited amyloid leads to increased resistance to venous return and retinal functional abnormalities. Therefore, careful follow-up of structural and functional changes in the retina is needed, even in asymptomatic patients with FAP.

## 1. Introduction

Familial amyloidotic polyneuropathy (FAP) comprises a group of autosomal dominant hereditary diseases, which are mostly caused by mutations in the protein transthyretin (TTR). First described in 1952, FAP is characterized by the initial involvement of peripheral nerves, especially in the lower extremities [1]. Variable clinical presentations occur between kindreds with the same mutation and even among family members [2]. Amyloid deposition of mutant TTR may cause lesions in various organs, including the peripheral nerves (sensorimotor polyneuropathy and autonomic dysfunction), the heart (restrictive cardiomyopathy and cardiac conduction disturbance), the central nervous system (dementia, stroke and cerebral hemorrhage), the kidney (renal insufficiency) and the eye [3]. The ocular abnormalities include abnormal conjunctival vessels, keratoconjunctivitis sicca, amyloid deposition in the anterior segments, glaucoma, vitreous opacities and retinal vascular lesions [4]. Liver transplantation [5,6] and oral administration of tafamidis, the TTR tetramer stabilizer [7,8], have been proven to stabilize or relieve the symptoms and increase the survival rates of patients with FAP. However, ocular manifestations, including vitreous opacities, may still occur after liver transplantation [9] or tafamidis treatment [10].

More than 100 genetic mutations in TTR were linked with FAP [11], with c.148G>A (p.Val50Met) being the most common mutation [12]. The amyloidogenic point mutation c.401A>G (p.Tyr134Cys), which involves the substitution of tyrosine with cysteine at codon 114, is a rare genotype, and it was first reported in two Japanese siblings in 1990 [13]. Patients with FAP carrying the c.401A>G (p.Tyr134Cys) mutation have a higher prevalence of vitreous opacities than patients with the c.148G>A (p.Val50Met) mutation [14]. Vascular lesions, such as retinal amyloid angiopathy and choroidal amyloid angiopathy, are also common in patients with the c.401A>G (p.Tyr134Cys) mutation [15]. Moreover, retinal vein occlusion (RVO) is not uncommon among patients with FAP, as in the patients with the c.401A>G (p.Tyr134Cys) mutation [16,17].

A postmortem study showed that amyloid deposition of mutant TTR in the heart was apparent even in asymptomatic patients [18]. Although typical vitreous opacities and angiopathies in patients with FAP are well documented, few reports described whether intraretinal amyloid deposition occurs before or after the appearance of vitreous amyloid. Furthermore, little is known about the pathogenic effects of amyloid deposition on the retinal structure and function. 

In this study, we evaluated five Chinese cases of FAP with the TTR c.401A>G (p.Tyr134Cys) mutation by using multimodal retinal imaging and electrophysiological examination. These cases were in various stages with different ocular manifestations. Importantly, we identified early retinal structural and functional changes even in patients with no ocular symptoms. We also found that the mutant TTR amyloid was deposited in the internal limiting membrane (ILM), accompanied by more intense staining of transforming growth factor (TGF)-β1.

## 2. Methods

### 2.1. Participants and Clinical Examination

**Figure 1:**
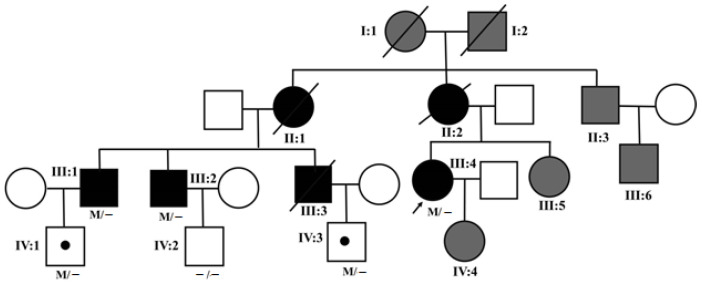
The Chinese pedigree of FAP. Arrow: the proband. Black symbols: affected individuals. Grey symbols: unidentified individuals. Slashed symbols: deceased individuals. Black dots: asymptomatic individuals with the mutation TTR c.401A>G. M: mutation TTR c.401A>G.

This study was conducted in accordance with the ethical standards stated in the Declaration of Helsinki and was approved by the IRB/Ethics Committee of Eye and ENT Hospital of Fudan University (No. KJ2009-16). Informed consent for the research was obtained from all participants in the study. A pedigree with FAP was recruited at the Eye and ENT Hospital of Fudan University, Shanghai, China (Figure 1).

In this pedigree, the members I:1, I:2, II:1, II:2 and III:3 were deceased, and the members II:3, III:5, III:6 and IV:4 did not report any symptoms and refused to participate in this study. The members III:1, III:2, III:4, IV:1, IV:2 and IV:3 were genotyped. IV:2 was unaffected, and the remaining five members, genetically diagnosed with FAP, underwent thorough ophthalmic examinations, including best-corrected visual acuity (BCVA), intraocular pressure (IOP), ocular B-scan ultrasonography, fundus photography (TRC-50DX, Topcon Inc., Tokyo, Japan), ultra-wide field photography and ultra-wide field angiography (UWFA) (Optos 200Tx, Optos Plc., Dunfermline, UK), optical coherence tomography (OCT) (Spectralis, Heidelberg Engineering Inc., Heidelberg, Germany), autofluorescence (AF), near-infrared AF, fluorescein angiography (FA), indocyanine green angiography (ICGA), optical coherence tomography angiography (OCTA) (RTVueXR AVANTI, Optovue, Fremont, CA, USA), full-field electroretinogram (ffERG) (UTAS visual diagnostic test system, LKC Technologies Inc., Gaithersburg, MD, USA) and multifocal electroretinography (mfERG) (Veris system 6.0.10, Electro-Diagnostic Imaging Inc., Burlingame, CA, USA). On FA, the arteriovenous passage time (AVT) was defined as the interval from the appearance of dye in the retinal arteries to the end of laminar flow in the major retinal veins in the corresponding quadrant. The normal AVT value is between 5 and 12 s [19]. The retinal nerve fiber layer (RNFL) thickness was also measured by OCTA in five healthy subjects.

### 2.2. Surgical Procedures

Eyes with significant vitreous opacities and reduced BCVA underwent 23-gauge pars plana vitrectomy (Constellation, Alcon Laboratories Inc., Fort Worth, TX, USA). Vitreous specimens were obtained for histological analysis. In case 1, the residual posterior hyaloid was tightly attached to the macula. The ILM was peeled to prevent postoperative epiretinal membrane formation. The macular ILM of a patient with an idiopathic macular hole (IMH) was obtained as a histopathological control.

### 2.3. Histological Analysis

Vitreous specimens were stained with hematoxylin and eosin (H&E) for light microscopic observation (Leica Microsystems, Wetzlar, Hesse-Darmstadt, Germany) and Congo red for both light and polarized microscopic observation. The macular ILMs obtained from case 1 and the patient with IMH were analyzed. After formalin fixation and embedding in paraffin, the samples were cut into 5-μm-thick slices. The slices were stained with H&E for light microscopy and then stained with Congo red for both light and polarized microscopy. For immunohistochemical staining, the slices were first blocked with 3% hydrogen peroxide for 25 min and then incubated with 10% goat serum for 30 min. The slices were incubated with the primary antibodies: rabbit anti-vascular endothelial growth factor (VEGF) (ab183100, Abcam, Cambridge, UK) and rabbit anti-TGF-β1 (ab9758, Abcam) at 4 °C overnight. After being incubated with a horseradish peroxidase-conjugated secondary antibody for 1 h at room temperature, the slices were stained with 3,3′-diaminobenzidine for color development, and the nuclei were counterstained. The slices were observed on light microscopy, and images were taken.

### 2.4. Genetic Analysis

Peripheral blood samples were collected from all participants, and DNA was extracted from whole blood using the FlexiGene DNA Kit (Qiagen, Hilden, Germany) according to the manufacturer’s protocols. Targeted next-generation sequencing was performed using a gene panel comprising 455 genes involved in common retinal dystrophies (Mygenostics, Shanghai, China). In order to acquire the probe sequences, we obtained the exon sequence and its flank ± 30 bp of the 455 genes from a reference human genome (GRCh37/hg19). On average, the mean coverage depth was more than 400×, and the coverage of the target region was ~99.9% using a sequencing system (Illumina, San Diego, CA, USA). The amplified genomic sequences were compared with the TTR reference sequence NM_000371.3. The obtained sequence data were analyzed as previously reported [20]. Sanger sequencing was performed to validate the candidate variants, and segregation analysis was performed with family members. Moreover, three-dimension structures of wildtype- and Tyr114Cys-TTR were generated with PyMOL (Molecular Graphics System, DeLano Scientific, San Carlos, CA, USA).

## 3. Results

### 3.1. Case 1: Symptomatic Vitreous Haze and Advanced Chorioretinal Lesions

**Figure 2:**
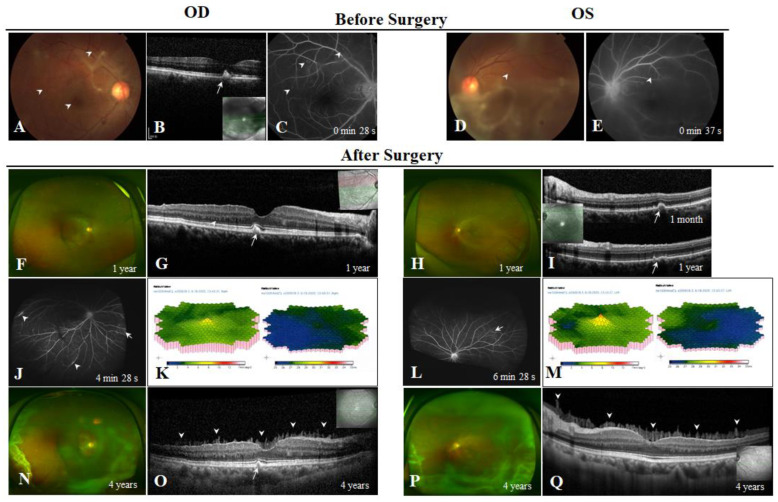
Case 1. (**A**,**D**) Fundus photographs of the right and left eyes before surgery, showing vitreous haze and yellowish spots (white arrowheads). (**B**) Optical coherence tomography (OCT) images of the fovea showing sub-retinal pigment epithelium (subRPE) hyperreflective deposit (white arrow). (**C**,**E**) Fluorescein angiography of the right and left eyes showed multiple hyperfluorescent spots (white arrowheads). (**F**,**H**) Postoperative fundus photographs of the right and left eyes. (**G**,**I**) Follow-up OCT of the left eye 1 year after surgery showed dissipation of the subRPE hyperreflective deposits (white arrows) and persistent intraretinal deposits in the outer retina (white arrowheads). (**J**,**L**) Ultra-wide field angiography 1 year after surgery, showing vascular sheathing, leakage (white arrowheads) and telangiectasis (white arrows). (**K**,**M**) Multifocal electroretinography showing bilaterally dampened amplitudes. (**N**,**P**) Fundus photographs of the right and left eyes 4 years after surgery show recurrent vitreous haze. (**O**,**Q**) OCT showed needle-shaped deposits adhering to the surface of retina (white arrowheads).

Case 1 (III:4) was a 44-year-old woman complaining of progressive bilateral floaters that persisted for several months. Her BCVAs were 20/125 OD and 20/200 OS when she first arrived at our hospital. Ophthalmic examination revealed vitreous opacities, which were more severe in the left eye (Figure 2A,D). Yellowish spots were found in the fundus of both eyes (Figure 2A,D, white arrowheads). These spots were sub-retinal pigment epithelium (subRPE) hyperreflective deposits on OCT (Figure 2B, white arrow) and were hyperfluorescent on FA (Figure 2C,E, white arrowheads). Both eyes underwent vitrectomy with peeling of the ILM. Her postoperative BCVAs increased to 20/40 OD and 20/32 OS (Figure 2F,H). The subRPE deposits dissipated during one year’s follow-up (Figure 2G,I,O, white arrows). Numerous hyperreflective amyloid deposits were found in the outer nuclear layer(ONL), outlining the ONL-outer plexiform layer (OPL) border with a dentate appearance (Figure 2G, white arrowheads). Ultra-wide field angiography (UWFA) showed vascular sheathing, leakage (Figure 2J, white arrowheads) and telangiectasis (Figure 2J,L, white arrows). FfERG showed normal bilateral scotopic and photopic responses; however, mfERG revealed bilateral reduced P1 amplitudes of the central area that were worse in the right eye (Figure 2K,M). The patient returned to our hospital 4 years after surgery due to recurrent vitreous opacities (Figure 2N,P). Her BCVAs decreased to 20/50 for both eyes. OCT revealed bilateral preretinal vitreous condensation and intraretinal amyloid deposits (Figure 2O,Q, white arrowheads). The IOP was normal in both eyes during the 4 years follow-up. Furthermore, there was no evidence of peripheral nerve or heart involvement.

### 3.2. Case 2: Symptomatic Vitreous Haze, Advanced Chorioretinal Lesions and Calcification and Atrophy of the Eyeball

**Figure 3:**
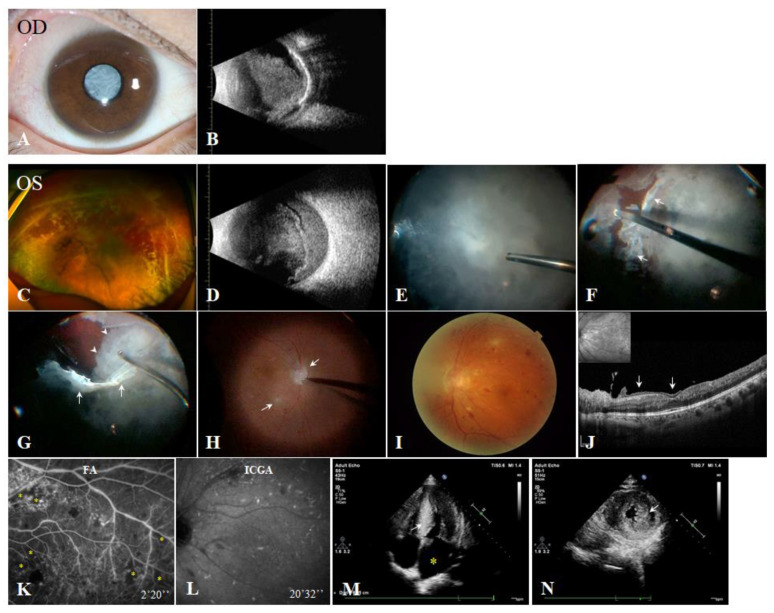
Case 2. (**A**) Anterior segment photograph of the right eye. (**B**) B-scan ultrasound image of the right eye, showing vitreous opacities and calcification of the eyeball wall. (**C**) Preoperative fundus photograph of the left eye, showing vitreous haze. (**D**) B-scan ultrasound image of the left eye, showing opacities in the vitreous cavity and preretinal space. (**E**–**H**) Intraoperative snapshot images showed cotton wool-like opacities (**E**), dense posterior hyaloids (**F**,**G**, white arrows), velvet deposits in the preretinal space (**G**, white arrowheads) and white deposits adhered to the optic disc and retina (**H**, white arrows). (**I**) Postoperative fundus photograph of the left eye, showing multiple hemorrhagic spots. (**J**) Postoperative optical coherence tomography image, showing deposits adhering to the surface of the retina and blurred retinal structures. (**K**) Fluorescein angiography of the left eye showed multiple areas of telangiectasis and nonperfusion. (**L**) Indocyanine green angiography of the left eye, showing hypercyanescence along the choroid vessels. (**M**) Apical four-chamber view of the heart on echocardiography, indicating left atrial enlargement and thickening of the ventricular septum. (**N**) Parasternal short-axis view at the level of the mitral valve, indicating hypertrophy of the left ventricular walls.

Case 2 (III:1) was the cousin of case 1. He was 40 years old and complained of progressive blurred vision in his left eye for over 2 years. When he came to our hospital, his BCVAs were light perception OD and 20/800 OS. His IOPs were 8 mmHg OD and 14 mmHg OS. He reported his right eye ‘went blind’ decades earlier for an unknown reason and was never treated. An ophthalmic examination revealed a cataract in his right eye (Figure 3A). B-scan ultrasonography showed widespread calcification of the eyeball wall (Figure 3B). His left eye had a normal anterior segment with significant vitreous opacities (Figure 3C). B-scan ultrasonography also revealed preexisting posterior vitreous detachment (PVD), and opacities were found in the vitreous, with denser opacities in the preretinal space (Figure 3D). His axial lengths were 22 mm OD and 26 mm OS. During the vitrectomy of the left eye, we found that the vitreous cavity contained numerous cotton wool-like amyloid opacities (Figure 3E), the posterior hyaloid had a reticular appearance (Figure 3F,G, white arrows), and the preretinal space was full of velvet amyloid deposits (Figure 3G, white arrowheads). Some amyloid deposits tightly adhered to the retina and the optic disc (Figure 3H, white arrows). The retinal veins were tortuous, and intraretinal hemorrhages and exudates were present (Figure 3H,I). One month after the vitrectomy, his vision increased slightly to 20/200 OS. OCT showed blurred retinal structures, intraretinal amyloid deposits and preretinal membrane-like vitreous condensation (Figure 3J, white arrows). FA revealed numerous microaneurysms, venous beading, vascular sheathing and leakage and significant capillary nonperfusion (Figure 3K, yellow asterisks). ICGA identified multiple late hypercyanescence along the choroid arteries (Figure 3L), similar to the focal pattern proposed by Antoine Rousseau et al. [21]. His left eye underwent retinal photocoagulation to prevent neovascularization. The IOP of the left eye was normal throughout the 1 year’s follow-up. Although he did not report any neurological or cardiac symptoms, preoperative examinations revealed a highly elevated pro-brain natriuretic peptide (proBNP) of 3006 pg/mL (normal range, <100 pg/mL) and dampening of the S-T segment on electrocardiogram (ECG). Echocardiography confirmed left atrial enlargement (Figure 3M, yellow asterisk) and thickening of ventricular septum and walls with a ground-glass appearance (Figure 3M,N, white arrows). The patient was suspected of having amyloid cardiomyopathy and was referred to the cardiology department.

### 3.3. Case 3: Unilateral Symptomatic Vitreous Haze and Bilateral Moderate Chorioretinal Lesions

**Figure 4:**
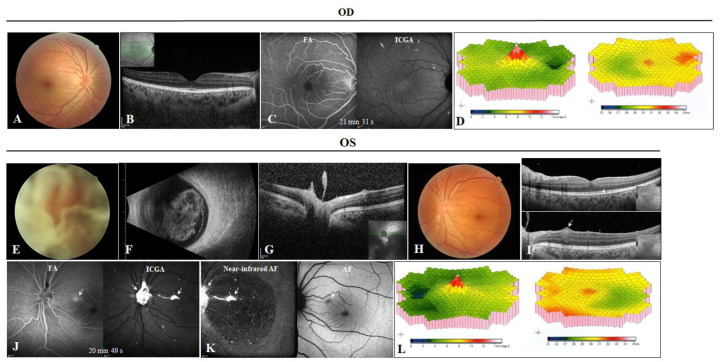
Case 3. (**A**) Fundus photograph of the right eye, showing tortuous veins. (**B**) Optical coherence tomography (OCT) image through the fovea of the right eye was mostly normal. (**E**) Preoperative fundus photograph of the left eye, showing vitreous haze. (**F**) B-scan ultrasound of the left eye. (**G**) Preoperative OCT image showing deposits on the surface of the optic disc. (**H**) Postoperative fundus photograph of the left eye, showing tortuous veins. (**I**) Postoperative OCT image showing flattening of the foveola as well as deposits in the retina (white arrowhead) and around the superficial vessels (white arrow). (**C**,**J**) Fluorescein angiography (FA) of both eyes showed a branch of a leaking artery in the left eye, while indocyanine green angiography of both eyes showed multiple hypercyanescence in both eyes and strong staining of the optic disc and the leaking artery (on FA). (**K**) Near-infrared autofluorescence and autofluorescence of the left eye. (**D,L**) Multifocal electroretinography showed bilateral decreased amplitudes and prolonged implicit times.

Case 3 (III:2) was a 43-year-old brother of case 2. He complained of decreased vision of the left eye for several months. His BCVAs were 20/20 OD and 20/400 OS. Retinal venous tortuosity was found in his right eye (Figure 4A). The macular structure of the right eye seemed normal (Figure 4B). His left eye had significant vitreous opacities (Figure 4E), confirmed by B-scan ultrasonography (Figure 4F). OCT revealed highly reflective opacities attached to the optic disc (Figure 4G). His left eye underwent a vitrectomy, and the BCVA improved to 20/20. Venous tortuosity was also seen (Figure 4H). Other than flattening of the foveola and a dentate appearance (Figure 4I, upper panel, white arrowheads), the retinal structures were normal on OCT. FA indicated tortuous veins in both eyes (Figure 4C,J, left panels) and early hyperfluorescence with late leakage from one parafoveal retinal artery in the left eye (Figure 4J, left panel, white arrow). The AVT was 12.80 s in the left eye. ICGA showed bilateral choroidal amyloid angiopathy and hypercyanescent amyloid residues attached to the optic disc and along the FA-leaking artery (Figure 4C,J, right panel, white arrow; Figure 4I, lower panel, white arrow). The amyloid residues were hyperautofluorescent on a near-infrared AF image but were hypoautofluorescent on an AF image (Figure 4K, white arrows). FfERG showed normal bilateral responses, but mfERG indicated bilateral decreased P1 amplitudes with prolonged P1 implicit times (Figure 4D,L). A follow-up of about half the year showed normal IOP in both eyes. There was no sign of peripheral nerve or heart involvement in this patient.

### 3.4. Case 4 and Case 5: Bilateral Asymptomatic Carriers and Mild Retinal Venous Tortuosity

**Figure 5:**
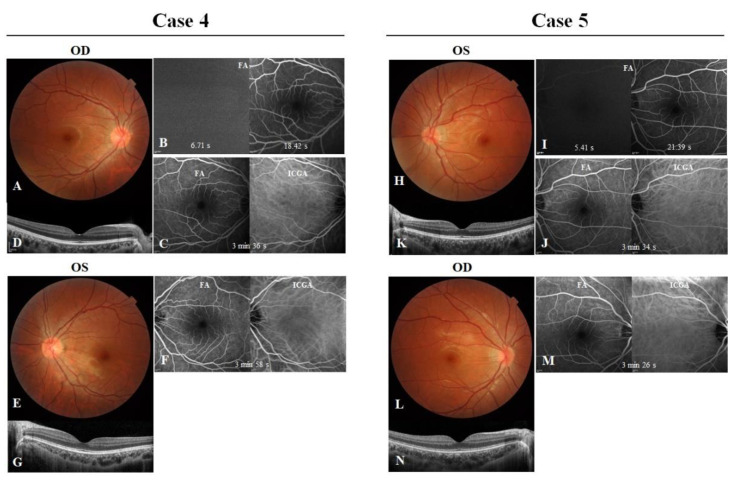
Cases 4 and 5. (**A**,**E**) In case 4, fundus photographs of the right and left eyes showed slightly tortuous veins. (**B**) Fluorescein angiography (FA) of the right eye showed that the dye appeared in the superiortemporal arteries at 6.71 s, and the major vein in the corresponding area was completely filled at 18.42 s. Optical coherence tomography (OCT) images (**D**,**G**), FA and indocyanine green angiography (ICGA) (**C**,**F**) of the right and left eyes showed no abnormalities. (**H**,**L**) In case 5, fundus photographs of the left and right eyes showed tortuous veins in the left eye. (**I**) FA of the left eye, showing that dye appeared at 5.41 s and venous filling was completed at 21.39 s. OCT images (**K**,**N**), FA and ICGA (**J**,**M**) of both eyes were apparently normal.

Case 4 (IV:1) was the 22-year-old nephew of cases 2 and 3, and case 5 (IV:3) was the 15-year-old son of case 2. Both cases had bilateral BCVAs of 20/20. Fundus examination revealed mild venous tortuosity in both eyes of case 4 (Figure 5A,E) and in the left eye of case 5 (Figure 5H). The AVTs were 11.71 s for the right eye of case 4 (Figure 5B) and 15.98 s for the left eye of case 5 (Figure 5I). The OCT, FA and ICGA tests revealed normal chorioretinal structures (Figure 5C,D,F,G,J,K,M,N). 

### 3.5. RNFL Analysis

**Figure 6:**
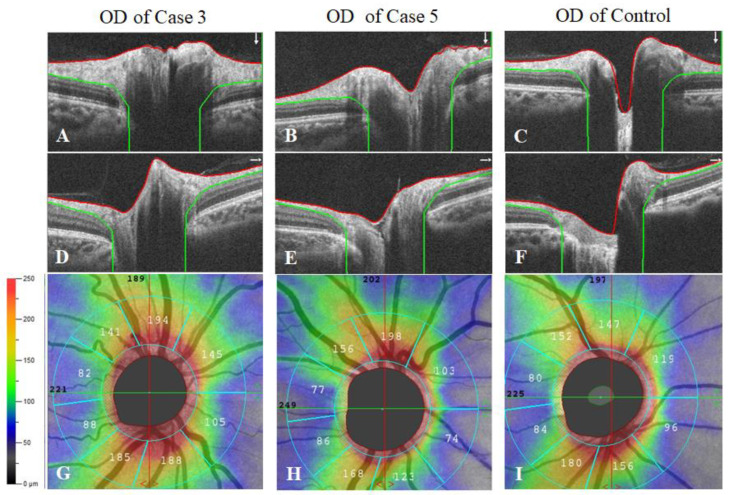
Measurement of the Retinal Nerve Fiber Layer (RNFL) Thickness. (**A**–**C**) Vertical scans of the right eyes from case 3, case 5 and the healthy control. (**D**–**F**) Horizontal scans of the right eyes from case 3, case 5 and the healthy control. (**G**–**I**) Representative optical coherence tomography angiography images showing the RNFL thickness around the optic disc in the right eyes from case 3, case 5 and the healthy control.

We examined the RNFL in cases 3–5 and compared the results with those of healthy controls. The RNFL was not assessed in cases 1 and 2 due to significant vitreous opacities and poor fixation, respectively. Cases 3–5 had smaller or even absent optic cups, and the optic discs with a crowded appearance (Figure 6A,B,D,E), compared with the healthy controls (Figure 6C,F). Moreover, RNFL in cases 3–5 were apparently thicker on average (130.3μm) than that in the healthy controls (118.7 μm) (Figure 6G–I). However, there were too few cases to make a statistical comparison.

The brief clinical characteristics of the family members included in this study are lisited in Table 1.

### 3.6. Histological Examination

**Figure 7:**
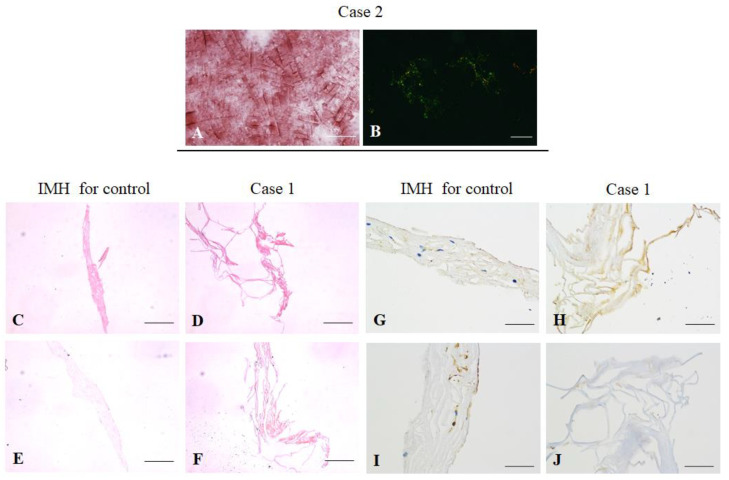
Histopathological examination. (**A**,**B**) Congo red staining of vitreous aspirates from case 2 under the light microscope and a characteristic apple-green birefringence under a polarized microscope. Scale bar: 200 μm. (**C**,**D**) Hematoxylin and eosin staining of the inner limiting membranes (ILM) from a patient with idiopathic macular hole (IMH) and case 1. Scale bar: 100 μm. (**E**,**F**) Positive Congo red staining of the ILM in case 1. Scale bar: 100 μm. (**G**,**H**) Immunohistochemical staining of ILM, showing increased transforming growth factor-β1 expression in the ILM from case 1. Scale bar: 50 μm. (**I**,**J**) Immunohistochemical staining of the ILM showed no difference in vascular endothelial growth factor between case 1 and the patient with IMH. Scale bar: 50 μm.

The vitreous samples obtained from cases 1 to 3 were eosinophilic on H&E staining, Congo red positive on light microscopy and showed apple-green birefringence on polarized microscopy (Figure 7A,B). ILM samples collected from case 1 and the patient with IMH were compared. Both samples were eosinophilic on H&E staining, but the Congo red stain was only positive in case 1 (Figure 7C–F). Prior studies have suggested that TGF-β1 expression is correlated with amyloid deposition and vascular degeneration in central nervous system diseases, including Alzheimer’s disease (AD) and cerebral amyloid angiopathy [22]. The staining of TGF-β1 was increased in the ILM of case 1, as compared with that in the patient with IMH (Figure 7G,H). There was no increase in the staining of VEGF, which plays a pivotal role in retinal vascular leakage and neovascularization, in either sample (Figure 7I,J)

### 3.7. Genetic Analysis

**Figure 8:**
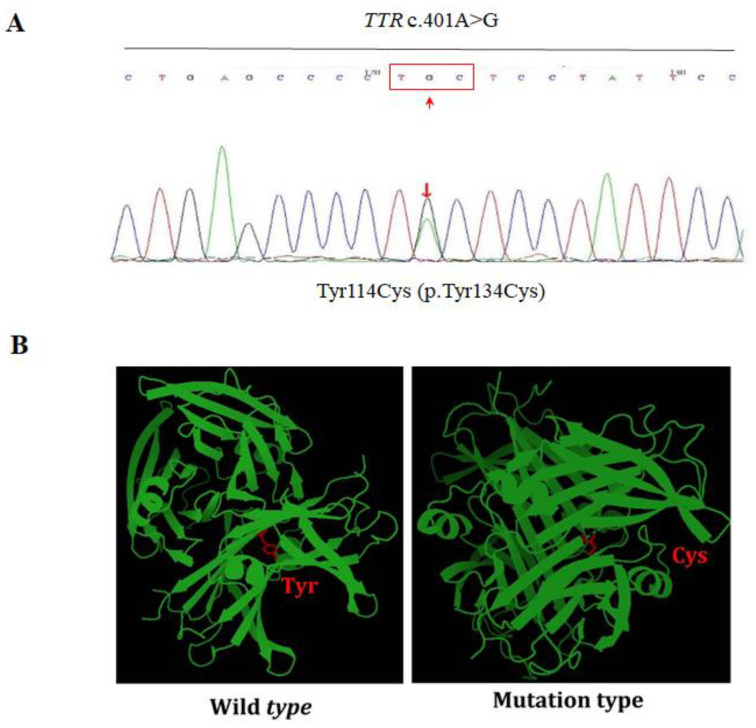
Genetic Analysis of the Mutation TTR c.401A>G (p.Tyr134Cys). (**A**) Representative image of genetic sequencing of TTR in all five cases, indicating transversion of adenosine to guanine at position 401, which resulted in substitution of tyrosine with cysteine at position 114 of exon 4. (**B**) Comparison of the three-dimension structures of wildtype and mutation type (Tyr114Cys) of TTR generated by PyMOL.

Gene analyses were performed for all cases and identified the same transversion of adenosine to guanine at base pair 401 in exon 4 (c. 401A>G) (Figure 8A). This variant causes the substitution of tyrosine with cysteine at codon 114 (Tyr114Cys). The numbering is based on the protein sequence of the mature TTR. Alternatively, the numbering based on the sequence of primary unprocessed TTR, which includes the signal peptide (20 amino acids), makes p.Tyr134Cys the synonym. The bioinformatics tool SIFT predicted the mutation is damaging. The three-dimension structures of wildtype and mutant TTR (Tyr114Cys) were also presented (Figure 8B), and the normal (Tyr) and mutant (Cys) residues in structures were marked in red.

## 4. Discussion

Patients with FAP carrying the mutation TTR c.401A>G (p.Tyr134Cys), first reported in Japan, manifested polyneuropathy, vitreous opacities and cardiopathy [13]. In this study, we presented the clinicopathological characteristics derived from multimodal retinal imaging and electrophysiological examination of five cases with the c.401A>G (p.Tyr134Cys) mutation in TTR. In addition to the characteristic vitreoretinal abnormalities and histological findings, we also observed early structural and functional retinal changes in the asymptomatic eyes in these five cases. 

TTR is a homo-tetramer, and each monomer is rich in βsheets. Monomers are linked to dimers by interactions between antiparallel β-sheets. The dimer-dimer interaction is based on a short loop of each monomer. TTR helps transport thyroxine (T4) and retinol in the bloodstream into tissues, including the retina. However, TTR null mice showed no abnormalities in retinal structure and function [23]. Various mutations of TTR cause its depolymerization, formation of amyloid fibrils and subsequent amyloid deposition in organs and tissues. Mutant TTR amyloid shows a high affinity for components of the extracellular matrix in the basal membrane, especially fibronectin [24]. Fibronectin in the vitreous interlinks vitreous collagen fibrils [25]. Therefore, TTR amyloid may adhere to the vitreous fibrils through fibronectin, explaining the cotton wool-like vitreous opacities and the reticular posterior hyaloids observed in case 2. Compared with the vitreous opacities, the preretinal velvet aggregates were looser and more mobile, probably due to a lack of fibronectin in the preretinal space after spontaneous PVD. Surprisingly, for the right eye of case 2, the relatively low IOP and the relatively short axial length compared with the left eye and widespread calcification of the eyeball wall indicated the sub-atrophy stage. It has not been reported in patients with FAP. We did not consider traumatic injuries, glaucoma or long-standing retinal detachment based on the patient’s medical history, IOP and B scan results. However, The severe retinal vascular damage in the left eye, including significant intraretinal hemorrhages and non-perfusion area, implies that the right eye might also suffer from the insufficient blood supply. We speculate ischemia may play an important role in the gradual atrophy of the affected eye.

RPE is the main source of TTR in the neurosensory retina [26]. Pan-retinal photocoagulation may injure the RPE and reduce the amyloid deposition [27]. The Bruch’s membrane is essentially the basal membrane which mutant TTR amyloid shows high affinity for [28]. We speculate that the subRPE deposits contained amyloid secreted by the RPE. Given amyloid can dissipate gradually, it may disperse into the neurosensory retina or the choroid. 

Our findings suggest that retinal amyloid deposits were present in these cases. On the one hand, we observed apparently thicker RNFL even in the asymptomatic cases. In a postmortem study of FAP patients carrying the V30G mutation, amyloid deposits were detected in the superficial retinal layers [29,30]. In our cases, we observed that amyloid deposits adhered to the surface of the optic disc during vitrectomy in cases 2 and 3. In case 3, OCT and near-infrared AF also verified amyloid deposition in the optic disc. The amyloid deposition may explain the apparently thicker RNFL and crowded optic disc. In addition, venous tortuosity was also visible in the symptomatic and asymptomatic eyes in case 3. Cases 4 and 5 also showed retinal venous tortuosity at a younger age. AVT values of cases 3, 4 and 5 were slightly prolonged (12.80 s), around the upper limit (11.71 s) and prolonged (15.98 s), respectively. Accordingly, the venous return resistances were mildly elevated, close to elevation and elevated in cases 3, 4 and 5, similar to the early stage of RVO. As previously reported [31], the drusen of the optic disc could predispose to RVO. Therefore, the venous changes, even in the asymptomatic stage, may be due to the mechanical compression from gradual amyloid deposition in the optic disc. Because RVO can be observed in patients with FAP [16], venous tortuosity and abnormal AVT should be regarded as risk factors for RVO, necessitating careful follow-up even in asymptomatic carriers.

On the other hand, intraretinal deposits in the outer retina were also observed in cases 1–3. This was demonstrated by the dentate borderline between the ONL and OPL, even in the right eye of case 3, with postoperative BCVA 20/20. Interestingly, although ffERG responses appeared normal, the abnormalities in mfERG P1 amplitude and implicit time were observed in cases 1 and 3. Aberrant ffERG responses were noticed in other patients with FAP, with an average age older than that in the present study [32]. MfERG is more sensitive in detecting foveal and/or macular dysfunction than ffERG [33], and it can indicate underlying retinal damage in apparently normal eyes [34]. In vitro studies have demonstrated the cytotoxicity of amyloid fibrils composed of mutant TTR (Tyr114Cys) [35]. Another amyloidogenic protein, β-amyloid (Aβ), also deposits in the retina, both in the transgenic mouse model of AD and patients with AD. Similarly, it also contributes to a decrease in retinal electrical activity, reflected by mfERG [36,37]. Thus we believe that intraretinal amyloidogenic proteins deposition, including mutant TTR in FAP and Aβ in AD, is both cytotoxic to the retina. Additionally, mfERG can serve as a sensitive tool to monitor the retinal dysfunction before clinical symptoms. 

Histopathological analysis revealed both amyloid deposition and more intense staining of TGF-β1 in the ILM. Previous studies showed that TGF-β1 increases the permeability of the vascular endothelium [38,39]. In AD, TGF-β1 is amyloidogenic, and its expression is positively correlated with the severity of cerebral amyloid angiopathy [22,40]. Therefore, TGF-β1 might be involved in retinal vascular lesions by inducing perivascular amyloid deposition or directly breaking down the blood–retina barrier. This possibility is supported by the observation of amyloid deposits along the leaking retinal vessel. However, the exact role of TGF-β1 in the pathogenesis of amyloidosis requires further investigation.

In conclusion, this study recorded five cases of FAP with the TTR mutation c.401A>G (p.Tyr134Cys) from one pedigree. These cases displayed different stages of vitreoretinal lesions. Aside from the vitreous opacities in the advanced stage, we also identified early structural and functional changes in the retina, even before clinical symptoms in cases with FAP.

## Figures and Tables

**Table 1:** Summary of the clinical information of the family members included in the study.

ID	Age (Year)	Gender	Diagnosis	Symptoms	Ocular Examination
III:1	40	Male	Affected	Light perception (OD); blurred vision (OS)	Sub-atrophy with cataract, vitreous opacities and calcification (OD); vitreous opacities, blurred retinal structure on OCT, retinal venous tortuosity with hemorrhage, severe vascular lesions on angiography (OS)
III:2	43	Male	Affected	Decreased vision (OS)	Vitreous opacities (OS), bilaterally increased thickness of RNFL, intraretinal amyloid deposits on OCT, retinal venous tortuosity, mild vascular lesions, increased AVT on angiography and aberrant mfERG responses
III:4	44	Female	Affected	Bilateral floaters	Vitreous opacities, intraretinal/subRPE amyloid deposits on OCT, severe vascular lesions on angiography and aberrant mfERG responses
IV:1	22	Male	Asymptomatic carrier	Normal	Increased thickness of RNFL, retinal venous tortuosity and AVT at the upper limit on FA
IV:2	22	Male	Unaffected	Normal	N/A
IV:3	15	Male	Asymptomatic carrier	Normal	Increased thickness of RNFL, retinal venous tortuosity and increased AVT on FA

## Data Availability

Data are available from the authors.
